# Damage control surgery: a new “way of thinking” in the treatment of the critically injuried

**DOI:** 10.1186/1471-2318-11-S1-A31

**Published:** 2011-08-24

**Authors:** Antonio Martino, Ciro De Martino, Gautam Maharajan, Marco Evangelista, Rosa Maria Giamattei, Anna Pisapia

**Affiliations:** 1 Casa di Cura “A. Grimaldi” di San Giorgio a Cremano (NA). Dipartimento di Chirurgia, Italy; 2 Università degli Studi di Napoli “Federico II”. U.O.C. di Chirurgia Generale, Italy

## Background

Damage control is well established as a potentially life-saving procedure in a few selected critically injured patients. In these patients the “lethal triad” of hypothermia, acidosis, and coagulopathy is presented as a vicious cycle that often cannot be interrupted and which marks the limit of the patient's ability to cope with the physiological consequences of injury.

The principles of damage control have led to improved survival and to the stopping of bleeding until the physiologic derangement has been restored and the patient could undergo a prolonged operation for definitive repair.

## Methods

There are five critical decision-making stages of damage control: I, patient selection and decision to perform damage control; II, operation and intraoperative reassessment of laparotomy; III, resuscitation in the intensive care unit; IV, definitive procedures after returning to the operating room; and V, abdominal wall reconstruction.

## Results

Although morbidity remains high, it is acceptable if it comes in exchange for improved survival. Damage control surgery offers a simple effective alternative to the traditional surgical management of complex or multiple injuries in critically injured patients. Phases I and II can be done at a rural hospital before transfer to a major trauma centre for definitive repair.

## Conclusions

There is a complex interplay between primary injury, particularly major abdominal injury in the multi-system trauma patient, and secondary injury, which relates to patient physiology, decision making and surgical technique. Analysis of outcomes is further confounded by the variety of surgical techniques used. The challenge is to match the correct operation, for a critically injured patient, with the patient's physiology. Excellence in general surgery does not equate with excellence in trauma surgery, and a clear understanding of damage control is essential.
